# What Is Homœopathy?

**Published:** 1885-10

**Authors:** J. T. Kent


### WHAT IS HOMŒOPATHY?

This question is a very broad one, and hence its answer cannot be limited or contracted. To say that Homœopathy is based upon the law of *similars* is but the bounding of a cone by describing its base and leaving its apex undiscovered and projecting into space; to say the least, the answer is unsatisfactory. When similars are mentioned, the novice immediately wonders what similars are referred to, and how are given similars related to each other. It is simple to affirm that similars nullify each other, and it is easy to demonstrate the fact, but other questions arise of greater importance and much harder to answer—how are these similars recognized, and how are they utilized to cure disease?

After hearing the statement that similars nullify each other, and having accepted the law expressed by the formula *similia similibus curantur*, what Homœopathy really is, is yet to be learned. The knowledge comes after due conversance with disease and drugs. One must acquire knowledge of disease in all its relations to the human body. One cannot afford to neglect any resource whereby he can gain information relative to disease. Causes, morbid anatomy, duration, and course of every disease in particular must be thoroughly studied. The habits of each and every fixed disease must be observed to acquire a knowledge of its true nature. One must be able to predict from the present what will likely take place in the immediate future. He must also know the sick-making substances and the sicknesses they produce, their course and duration, beginning and termination. From these the homœopathist arranges his similars. These are his *media* through which he develops a knowledge of the art of curing homœopathically. Without a careful and thoughtful study of the two, he can never answer the question which has been selected as the subject for this paper.

If he neglects a part he is ever crippled and in darkness as to the whole or totality. If he neglects to study disease in any of its many sides, he gropes in darkness during his lazy, half-useful life. If he reads morbid anatomy, and attempts to apply remedies by such knowledge, he must live and die with a life filled with numerous failures. The man who reads his symtomatology, as found in drug pathogenses, may do fine work, but he has neglected the half that he should have learned. The human body, the house of both health and sickness, must be searched until familiarity breeds contempt.

Homœopathy is the science of healing based upon the law of similars as a law of selection. To select under this *law*, one must be acquainted with parts and counterparts, positives and negatives—*similars*—that his *conclusions* may be made by *exclusion*, that he may demonstrate to himself as well that remedies are not indicated, as that the one similar only can conform to the disease in hand; appropriate, because it of all the known medicines is most like unto the disease to be cured. It is well known that many want to be called homœopathic physicians. Some desire the appellation who in practice have not this information mentioned above. They are not even acquainted with sick pictures. They only recognize disease in parts, not seeing the whole. These men alternate, or practice, by using a part of the picture of one drug and a part of the picture of another drug to cover the two portions of a supposed disease which they see only in a fragmentary state; not being acquainted with disease in totality, they cannot shape a picture in a single drug to fit any but the fragmentary disease. Only a few days ago one of these men said to me: "I have just prescribed Arsenicum and Sulphur on the pathology of the case." Being anxious to learn the pathology that furnishes such an infallible guide to these remedies, I made a pressing inquiry, but that which I learned was so vague I am unable to comprehend it.

The study of true pathology should be encouraged, and is essential to the science of Homœopathy, and no homœopathician has ever discouraged it. Pathology is any discourse upon disease; it is broad and all-embracing. The study of disease as manifested through subjective and objective symptoms, a study of lesions or results of disease as made known by physical inspection, etc., etc., down to morbid anatomy, all should be known by the homœopathician, with a full appreciation of the true value of all. The disease in its course, history, and every known manifestation should be considered that the individuality may appear in one grand picture.

Not until this picture, this totality, this individuality, is clear in mind, is grasped completely, can the physician deal with it intelligently; he will then see, in some pathogenesis, a picture with a similar totality and individuality standing out with the same bold relief. Now if he is acquainted with both, and acquainted with the grand law of selection expressed in *simila similibus curantur*, he will administer the medicine possessing in its pathogenesis this likeness of effect, and wait with the confidence peculiar to the experienced homœopathician. These are the primary and essential tenets of Homœopathy. The rest of the science is made up of degrees that perfect as they advance, and are qualitative in character and quantitative in appearance. Under these degrees we learn to play upon the strings of a vital harp with a *tactus eruditus*.

The next advancement deals with dynamization. Many are satisfied with the primary tenets of Homœopathy and want no more. They do not wish further instruction. They do not wish to be made conversant with the fact that all non-surgical diseases are dynamic in character (cause), and must be cured, even are cured *only*, by dynamic effects. They lose confidence in the potency of *Aurum* when it becomes too attenuate to guarantee visible gold, and yet they know that visible gold cannot be appropriated by a living stomach. Dynamic power begins to evolve very low in the scale of potentization, and may be evolved from the crude substance of some drugs. Experience, not philosophy, can satisfy the hungry mind as to the truth of this grandest achievement of the immortal Hahnemann.

When fully convinced that the dynamic power cures, another advancement awaits the student. He is then presented to the mysteries of dealing with the automatic forces of the living body when influenced by disease. He observes the effect of a dose of potentized medicine selected by the law of similars. It is indeed a small part of his observation to see the patient recover with no medicine but that contained in the dynamized drug. For greater things remain to be seen and studied. The aggravations and ameliorations found in peculiar diseased states are not so simple. The distress that may arise from a single dose of Sulphur in the last stage of phthisis is most astonishing; and the beginner cannot convince himself that the potentized drug was the cause of it. When I say to my class, You must not give Sulph. to the patient in the last stage of consumption, they all look at me in surprise. It is often observed that Phosphorus does great harm to low forms of organic disease. I have several times known a chronic invalid to go on with little suffering for a long time, and, with a hope to stay the progress of her disease, administered a single dose of a very high potency of an antipsoric medicine, only to distress her, put her in bed, and from which time her downward course was rapid, while I am convinced that had I avoided antipsorics she would have lived and suffered much longer. If a carefully selected antipsoric aggravates a low form of disease sharply, and the aggravation is protracted and no amelioration of the general condition follow, no more antipsorics should be thought of for that patient; the hope of cure must be abandoned, and short-acting medicines resorted to to palliate. In gout, cancer, phthisis, and organic diseases of this kind generally, the rule holds good. Any physician who has followed the use of high potencies for a considerable time must feel it. Then who can say there is no power developed? Only he who has not found this method of treating the sick. The physician that sees not these aggravations only demonstrates that he has made few or no homœopathic prescriptions. The closer the homœopathic relation between the remedy and disease, providing the disease is of low origin and well advanced, providing the disease is incurable, the sharper and more distressing will be the aggravation.

Once a fleshy, robust-looking lady, came into my office for professional aid; she looked so well that I suspected only a slight illness. Finally, a close study of her symptoms revealed the history of rheumatism, endocarditis, suffocation, amenorrhœa of eight months' duration, and great bodily suffering. Indeed, I was surprised that she manifested so little of her suffering. I compared her symptoms closely, and found that no remedy but Pulsatilla could correspond to her symptoms. This remedy was administered (51 M, Fincke), dry, one small dose, and Sac. Lac. She went home and felt very badly. Pelvic symptoms became marked, and she sent for me. She believed her flow would resume, and I hoped from her report that I had made a homœopathic prescription. But she struggled on and no flow appeared; her pelvic symptoms were such as should accompany her menstral *nisus*, but greatly intensified. I dare not repeat; success depended upon permitting the remedy to have its own way. She was made as comfortable as possible, and I waited on the remedy during this struggle for one or two weeks. The endocarditis then began to show itself with all its terrors, dark blood began to well up from the lungs, which grew worse from day to day, pulmonary œdema became marked, and blood-spitting increased from day to day. I felt that I must interfere and make an effort to save her life. The only result of the remedies selected was simply palliative. She passed away quietly.

I have treated several cases of gouty rheumatism in which I could plainly see that every dose of medicine advanced the original malady. Many times I have been forced to feel that the dose of a dynamized drug added new force to the old disease, and it progressed even more rapidly. I never saw such striking results from low attenuations. Not long ago I was called to the bedside of a patient in the last stage of phthisis. She had a diarrhœa, and passed large quantities of colorless urine; other symptoms accorded, and she took a dose of Acetic acid, which controlled the diarrhœa and polyurea, but immediately her chest symptoms came on with greater force than I was able to control, and she sank rapidly. I am sure she would have lived much longer had I permitted the less harmful conditions to go on. These things look strangely to the inexperienced physician, but they are facts; and, above all, show the great power of our potentized remedies. The truly appropriate remedy commonly develops the evidence of extreme sensitiveness in all kinds of sickness, and the extreme danger of repeating remedies is here illustrated.

If there is anything I dread it is an incurable disease. My experience in this line has been greater than I could ask. While these things have shown the danger of repeating medicines, they have also taught me another thing; viz.: I am generally able to predict the gravity of the disease by the manner of reaction that follows my remedy. In acute diseases I have not seen troublesome aggravations, but a pleasant increase of the existing symptoms or even new symptoms appearing is presumptive evidence of a good selection. In the western country our diseases are so mixed with that unknown quantity, or something that we call *malaria*, it is necessary to repeat medicines oftener in acute disease than in most countries. Malarial diseases and states are so cumulative in character that the effect of a single dose is soon exhausted and another becomes necessary. Therefore I find myself repeating frequently in many very acute cases. I begin by repeating once in two hours in a fever that is continued, but as soon as I see signs of a remission I stop all medicine and wait on Sac. Lac. When a fever is going up I repeat, and the instant it has ceased rising, I cease medicine. In agues I generally administer one or two doses in the apyrexia and wait results. I seldom administer medicine until the paroxysm has been completed. When the first dose is followed by a perceptible aggravation, a second dose should never be administered until the amelioration, which follows the aggravation, has ceased. When a medicine aggravates it will generally influence the patient much longer than when no such aggravation has been observed. An amelioration that begins forthwith also demands that all medicine be stopped, but such amelioration is seldom so striking as when the amelioration has been preceded by a slight aggravation. *Immediate amelioration* often indicates the *absence* of a deep-seated disease. Especially is this the case with the use of long-acting medicines. These go so deeply into the life that they shake the very foundation of the automatic existence. When these powers are so clearly demonstrated, can any man desire Morphine to quiet a patient in any kind of agony? Can any man feel the need of greater force to combat disease with?

Yes, there are men who do not know this force; it cannot be evolved at will by anybody who wills to evolve it. This force is never observed, except by him who has learned the philosophy taught in the *Organon* of Samuel Hahnemann; and it is after, *not before*, looking upon the wonderful effect of a remedy conforming to the law of similars that one can appreciate the power he has with which to combat the ills of life, and with which to defend frail man against the assaults of his natural enemy.

Then to the question, What is Homœopathy? I must answer, *no man knows! God* only knows the length and breadth of the intricate, unfathomable mystery. The knowable part of this science, if I may use the word, consists in observing the sick-making phenomena of drugs and the phenomena of sickness, gathering and grouping the similars, selecting with the likeness in view and waiting for results.

While we are observing the folly of others we must learn to avoid extremes in our own midst. We must not despise the original thirtieths of the master because we have found the Cm in so many cases so useful. While reveling in the higher degrees of the true healing art, the younger and weaker must be fostered while tremblingly climbling the pathway up the hillside so familiar to most of us. While the way is beset with thorns, it is nevertheless the way of truth, and no part of it is to be despised. With the young and old our faith must be pinned to the *law of similars, the single remedy, the smallest dose, the dynamic power*, and the last, but not least, the *proved drug*. These coupled with our organic philosophy, we shall continue in doing good and living to do good. J. T. Kent.

